# Serotypes and Genotypes of Invasive *Streptococcus pneumoniae* Before and After PCV10 Implementation in Southern Brazil

**DOI:** 10.1371/journal.pone.0111129

**Published:** 2014-10-30

**Authors:** Juliana Caierão, Paulina Hawkins, Fernando Hayashi Sant’anna, Gabriela Rosa da Cunha, Pedro Alves d’Azevedo, Lesley McGee, Cícero Dias

**Affiliations:** 1 Federal University of Health Science of Porto Alegre, Rio Grande do Sul, Brazil; 2 Emory University, Atlanta, Georgia, United States of America; 3 Centers for Disease Control and Prevention, Atlanta, Georgia, United States of America; Rockefeller University, United States of America

## Abstract

To reduce the burden of pneumococcal diseases, different formulations of pneumococcal conjugate vaccines (PCV) have been introduced in many countries. In Brazil, PCV10 has been available since 2010. We aimed to analyze the serotype and genetic composition of invasive pneumococci from Brazil in pre- and post- vaccination periods (2007–2012). Antibiotic susceptibility was determined and genotypes of macrolide and fluoroquinolone resistance were characterized. The genotypes of isolates of the most frequent serotypes were determined by multilocus sequence typing. The study included 325 isolates, which were primarily recovered from blood. The most common serotypes recovered were 14, 3, 4, 23F, 7F, 9V, 12F, 20, 19F, 8, 19A, and 5. Thirty-eight pneumococci (11.7%) were from children ≤5 years old. Considering the overall population, PCV10 and PCV13 serotype coverage was 50.1% and 64.9%, respectively. During the pre-vaccine period, isolates with serotypes belonging to the PVC10 represented 51.5% (100/194), whereas in the post vaccine they represented 48.0% (63/131). PCV13 serotypes represented 67.5% (131/194) and 59.2% (77/131) of total for pre- and post-vaccination periods, respectively. Seventy different sequence types [STs] were found, accounting for 9 clonal complexes [CCs] and 45 singletons. Eight STs (156, 180, 218, 8889, 53, 191, 770, and 4967) represented the majority (51.5%) of isolates. Fifty STs were associated with the pre-vaccination period (27 exclusive) and 43 (20 exclusive) with the post-vaccination period; 23 STs were identified in both periods. Some serotypes were particularly clonal (7F, 8, 12F, 20). Non-susceptibility to penicillin was associated with serotype 19A, CC320. Erythromycin resistance was heterogeneous when considering serotype and ST. A single serotype 23F (ST4967) isolate was resistant to levofloxacin. Continued surveillance is required to determine vaccine impact and to monitor changes in pneumococcal population biology post-PCV10 introduction in Brazil.

## Introduction

Infections associated with *Streptococcus pneumoniae* are a global public health problem. Invasive pneumococcal diseases (IPD), such as meningitis and bacteremia, are especially associated with high morbidity and mortality [1]. About 3,600 cases of meningitis, 14,000 cases of bacteremia, and between 200,000 and 300,000 cases of pneumonia associated with pneumococci occur annually in Latin America [2]. In Brazil, from 2000 to 2008, pneumococcal meningitis represented 11% of all bacterial meningitis, with an incidence of 9.5 cases/100,000 inhabitants in patients less than one year of age. Official data from Brazil demonstrate that in the same period 7,129,291 hospitalizations were associated with complications of community-acquired pneumonia where *S. pneumoniae* was the major etiological agent (www.datasus.gov.br). In addition, Andrade and co-workers estimated the incidence of IPD in children less than three years of age from Brazil (2007–2009) at 57.5/100,000 inhabitants [3].

To reduce the burden of pneumococcal disease, different formulations of pneumococcal conjugate vaccines have been introduced in many countries since 2000. In Brazil, a 10-valent formulation (PCV10) has been available for children less than 2 years of age since 2010 as part of the National Childhood Immunization Program, which is funded by Brazilian Federal Government. Although the benefits of PCV vaccination have been well documented in the countries in which it has been introduced [4], [5], [6], [7], [8], [9], [10], [11], [12], [13], [14], the success of the vaccine is influenced by the pneumococcal population, particularly serotype distribution in both colonization and invasive disease [15]. Indeed, while IPD associated with vaccine serotypes decreased significantly, some investigators detected a worrisome increase of IPD caused by serotypes not included in vaccine formulas [10], [15], [16].

Monitoring the epidemiology and understanding the dynamics of the pneumococcal population are essential to evaluating the efficacy of vaccines in each region, as well as indicating any need to modify vaccine formulations. In Brazil, a well-established program monitors the distribution of serotypes in invasive infections [17]; however, published studies additionally providing genotypes of *S. pneumoniae* are limited, especially for IPD. In the present study, we determined serotypes and genotypes of invasive pneumococci isolated from South Brazilian hospitals in periods pre- and post- vaccination (2007–2012) to identify possible vaccination- induced changes in the pneumococcal population.

## Materials and Methods

### Study setting and design

This is a retrospective study with *S. pneumoniae* recovered from patients with IPD attending three different hospitals (including two of the major tertiary hospital complexes of the city) in Porto Alegre, South Brazil, from 2007 to 2012. Porto Alegre has around 4 million inhabitants in its metropolitan area, and it is a major regional reference center for healthcare. Vaccination was introduced in Porto Alegre in August 2010, in a three-dose scheme (2, 4 and 6 months) plus a booster at 12 months. Catch-up campaign at time of introduction was as follows: 4 doses for aged 3–7 months; 3 doses for aged 8–9 months; 2 doses for aged 10–11 months; 1 dose for aged 12–23 months. In this context, pre- and post-vaccination periods in this study were considered years 2007–2010 and 2011–2012, respectively. Few data are available about PCV10 uptake in our region. Results of a recent study [14] demonstrated that from 2010 to 2011 PCV10 uptake in Porto Alegre was around 80%.

### Microbiological identification, serotyping and antimicrobial susceptibility

Isolates were maintained at −80°C and their identification was confirmed by standard methods: Gram stain, colony morphology, optochin susceptibility and bile solubility [18]. Serotypes were determined by Quellung reaction, using pool, type and factor-specific antisera at the Centers for Disease Control and Prevention (CDC), Atlanta, USA. Broth microdilution was used to determine Minimal Inhibitory Concentration (MIC). The following antimicrobials were evaluated: penicillin, ceftriaxone, vancomycin, meropenem, erythromycin, levofloxacin, tetracycline, clindamycin and trimethoprim-sulfamethoxazole. Results were interpreted according to the Clinical and Laboratory Standards Institute [19]. The reference strain *S. pneumoniae* ATCC 49619 was used for quality control.

### Genetic analysis of macrolide and quinolone resistance

Isolates presenting MIC≥0.5 µg/mL for erythromycin were submitted to a duplex PCR method for the detection of *erm*B and mef(A), according to Widdowson & Klugman (1998) [20]. To determine mutations present in pneumococci with levofloxacin MIC≥4 µg/mL, quinolone resistant determinant regions (QRDRs) of *gyr*A and *parC* were amplified and sequenced as described elsewhere [21].

### Molecular typing

Multilocus sequence typing (MLST) was performed according to adapted method described by Enright & Spratt (1998) [22] and detailed at http://www.cdc.gov/ncidod/biotech/strep/alt-MLST-primers.htm for the most frequent and/or relevant serotypes. Allele sequences were edited and complementary sense and antisense fragments were aligned using CodonCode Aligner. Allele profile and Sequence Type (ST) were obtained from the database of the MLST web site (http://pubmlst.org/spneumoniae/). In cases of new allele or ST, data were submitted for the approval of the curator before being published. eBURST groups were defined using the most stringent (conservative) definition of eBURSTv3: all ST assigned to the same group must share identical alleles at 7/7 or 6 of the 7 loci with at least one other member of the group. For clonal complexes (CCs), a cut-off point of five identical loci to the predicted founder of its eBURST group was used.

### Statistical and diversity analysis

Statistical and diversity analysis were performed using PAST software (version 2.17c). Simpson’s index of diversity and Shannon index were calculated in order to estimate diversity. The significance of the difference of the diversity indices between two groups was assessed by bootstrapping, as described in the PAST manual. The odds ratio (OR) for each specific serotype, compared to the total number of isolates, was determined; 95% confidence intervals (CIs) were calculated. P values<0.05 were considered to be statistically significant.

### Ethical considerations

This retrospective study was approved by the Research Ethics Committee of the Grupo Hospitalar Conceição (Project number 11–205), recognized by Comissão Nacional de Ética em Pesquisa (CONEPE) and by the Office for Human Research Protection (OHRP/USDHHS). No clinical data of patients were used in this study (informed consent not applied). Patient records/information was anonymized and de-identified prior to analysis.

## Results

A total of 325 non-duplicate pneumococci recovered from patients with IPD were included in the study. The most common isolation site was blood (n = 255, 78.5%), followed by cerebrospinal fluid (n = 54, 16.6%), pleural fluid (n = 12, 3.7%), ascites (n = 2, 0.6%), peritoneal fluid (n = 1, 0.3%) and joint fluid (n = 1, 0.3%). Patient age ranged from 0 to 94 years old, with an average of 45.2 years: 29 (8.9%), 38 (11.7%) and 72 (22.2%) were ≤2, ≤5 (0 to 60 months) and ≥65 years old, respectively. No age records were found for 6 patients and for two other no specific age was reported, although they were identified as “pediatric”. Among all isolates, 194 (59.7%) were recovered in the pre-vaccination period (2007–2010) and 131 (40.3%) were recovered post-vaccine introduction (2011–2012).

Forty serotypes were identified in total. *S. pneumoniae* infections were mostly associated with the following serotypes: 14 (11.4%), 3 (8.3%), 4 (7.1%), 23F (6.1%), 7F (5.8%), 9V (4.9%), 12F (4.9%), 20 (4.9%), 19F (4%), 8 (4%), 19A (3.4%) and 5 (3.4%). Three pneumococci were non-typable by Quellung reaction. Serotypes 10A, 13, 15A, 15B/C, 17F, 23B, 29, 33F and 35A were only found in the pre-vaccine period, and 7C, 9A, 28A and 38 were only found post-vaccine.

**Figure 1:**
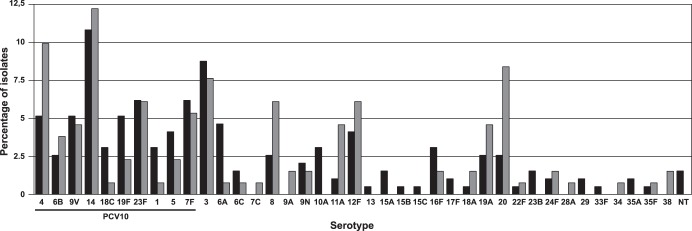
Distribution of serotypes in pre- and post-vaccination period among pneumococci recovered from IPD, during 2007–2012. Black bars represent pre-PCV10 and gray bars post-PCV10.

Figure 1 shows the distribution of serotypes before and after PCV10 introduction. It was observed some increase or decrease in the frequency of specific serotypes (Table S1). However, none was statistically significant, not even in the vaccinated population (children less than 2 years old).

Overall, PCV10 coverage was 50.1% and there was no statistical significance comparing PCV10 coverage before and after vaccination: 51.5% and 48.1%, respectively (p = 0.5411). Table 1 presents the occurrence of each PCV10 serotype before and after vaccination and Table 2 shows the PCV10 coverage over the years of the study, considering epidemiologically relevant age groups. PCV13 coverage among the general population was 64.9%.

**Table 1:** Distribution of serotypes belonging to PCV10 among invasive pneumococci recovered from 2007 to 2012 (the distribution of all serotypes is demonstrated on the Table S1).

Serotype	N (%)	Pre*	Post**	OR#	95% CI	P-value
14	37 (11.4)	21	16	0.7992	0.3998–1.5976	0.5260
4	23 (7.1)	10	13	0.4534	0.1925–1.0682	0.0704
23F	20 (6.2)	12	8	0.9335	0.3706–2.3516	0.8840
7F	19 (5.8)	12	7	1.0760	0.4119–2.8106	0.8812
9V	16 (4.9)	10	6	1.0439	0.3698–2.9467	0.9354
19F	13 (4.0)	10	3	2.1404	0.5774–7.9334	0.2549
5	11 (3.4)	8	3	1.6944	0.4409–6.5113	0.4426
6B	10 (3.1)	5	5	0.6154	0.1745–2.1701	0.4502
18C	7 (2.0)	6	1	3.8351	0.4562–32.2393	0.2159
1	7 (2.0)	6	1	3.8351	0.2265–6.9555	0.2159
**Total**	**163**	**100**	**63**			

*Pre-vaccination period;

**post-vaccination period;

# OR: odds ratio.

**Table 2:** PCV10 coverage over the years of the study (2007–2012), considering epidemiologically relevant age groups.

year	Age (n\%)	PCV10	Non-PCV10	p-value
2007	All (40; 100%)	27	13	
	≤5 years old (3; 7.5%)	2	1	0.974
	≥6 years old (37; 92.5%)	25	12	
2008	All (10; 100%)	3	7	
	≤5 years old (1; 10%)	0	1	0.570
	≥6 years old (8; 80%)	2	6	
	Unknown age (1; 10%)	1	0	
2009	All (72; 100%)	32	40	
	≤5 years old (6; 9.72%)	3	3	0.645
	≥6 years old (62; 86.1%)	25	37	
	Unknown age* (4; 4.16%)	4	0	
2010	All (23; 100%)	10	13	
	≤5 years old (5; 26.1%)	3	2	0.374
	≥6 years old (16; 69.6%)	6	10	
	Unkown age* (2; 4.34%)	1	1	
2011	All (119; 100%)	54	65	
	≤5 years old (18; 15.0%)	12	6	0.043
	≥6 years old (100; 84.8%)	41	59	
	Unknown age (1; 0.8%)	1	0	
2012	All (61; 100%)	33	28	
	≤5 years old (5; 8.19%)	1	4	0.110
	≥6 years old (56; 91,8%	32	24	
Total	All (325; 100%)	159	166	
	≤5 years old (38; 11.7%)	21	17	0.336
	≥6 years old (279; 85.8%)	131	148	
	Unknown age* (8; 2.5%)	7	1	

*included one isolate each year (2009 and 2010) recovered from a patient identified as “pediatric”: serotype 14 for both year.

To better determine if serotype distribution changed after PCV10 introduction, we compared pre (2009) and post-PCV10 (2012) periods. In 2009, among 72 isolates, 33 belonged to PCV10 serotypes (45.8%), while among 61 pneumococci recovered in 2012, 30 were from serotypes included in PCV10 (50.8%), which was not a statistically significant difference (p = 0.566).

Stratifying our population by age, PCV10 and PCV13 coverage was 55.3% and 73.7% among children ≤5 years old, respectively; and 49.4% and 63.8% among those >5 years of age, respectively. The occurrence of PCV10 serotypes did not vary statistically in pre- and post-vaccination era, neither among children under 5 years old (52.4% vs 58%), nor under 2 years old (58.8% vs 58.3%).

**Figure 2:**
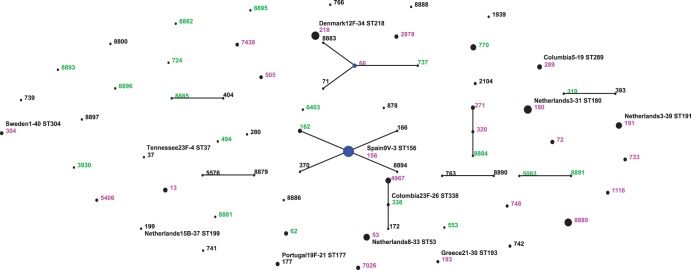
Population structure of invasive *S. pneumoniae* collected from 2007 to 2012. Black circles: pre-vaccine isolates; Green circles: post-vaccine isolates; pink circles: isolates present in both pre and post vaccine years; Blue circles: the ancestor of the CC.

Two-hundred thirty-one pneumococci, belonging to the 33 most relevant and/or frequent serotypes, were characterized by MLST. Our population was composed of 70 different STs, with 9 CCs (composed of 25 STs) and 45 singletons (Table 3; Figure 2). Eight STs (156, 180, 218, 8889, 53, 191, 770 and 4967) represented 51.5% (119/231) of all isolates. Most serotypes were associated with more than one ST and a few STs were associated with more than one serotype (Table 3): ST72 (3 and 24F), ST156 (14, 9V and 9A), ST218 (12F and 7F), ST733 (16F, 19A and 19F), ST4967 (19A and 23F) and ST5406 (23F and 9A). Fifty STs were found in the pre-vaccination period (with 27 exclusively related to this period), while 43 STs (20 exclusive) were recovered from IPD after PCV10 implementation; 23 STs were identified in both periods (Table 4). Considering Simpson’s index of diversity (94.8% and 94.9%, for pre and post, respectively) and Shannon index (3.47 and 3.33 before and after, respectively), genetic diversity was similar in both periods (p = 0.996 and p = 0.348 for Simpson’s and Shannon indexes, respectively), which is also true for PCV10 (32 STs identified) versus non-PCV10 (43 STs characterized) serotypes (Simpson’s index: 89.44% vs 91.4%; p = 0.189).

**Table 3:** Serotypes and STs observed among 231 pneumococci recovered from IPD (2007–2012).

Serotype (n)	ST dominant (n)	Other STs (n)
**14 (31)**	156 (22)	13 (5), 166 (1), 370 (1), 8881 (1), 8894 (1)
**3 (24)**	180 (17)	505 (3), 1176 (3), 72 (1)
**12F (15)**	218 (15)	
**20 (15)**	8889 (15)	
**4 (14)**	770 (9)	7026 (4), 8893 (1),
**9V (13)**	156 (9)	162 (4)
**8 (13)**	53 (11)	404 (1), 8885 (1)
**19F (12)**	271 (4), 177 (4)	733 (1), 763 (1), 878 (1), 8890 (1)
**23F (12)**	4967 (7)	338 (2), 37 (1), 5406 (1), 8895 (1)
**7F (12)**	191 (10)	218 (2)
**19A (11)**	2878 (4)	320 (2), 199 (1), 733 (1), 4967 (1), 8800 (1), 8884 (1)
**6B (6)**	748 (2)	553 (1), 724 (1), 8897 (1), 8896 (1)
**5 (6)**	289 (6)	
**11A (5)**	62 (4)	8882 (1)
**9N (5)**	66 (4)	8883 (1)
**6A (4)**		1939 (1), 8888 (1), 8879 (1), 5576 (1)
**16F (4)**	7438 (3)	733 (1)
**1 (3)**	304 (3)	
**18C (3)**	193 (2)	280 (1)
**24F (3)**	72 (3)	
**10A (3)**	742 (2)	741 (1)
**38 (2)**		393 (1), 310 (1)
**18A (2)**		8891 (1), 5063 (1)
**35A (2)**	2104 (2)	
**6C (2)**		172 (1), 3930 (1)
**9A (2)**		156 (1), 5406 (1)
**15B (1)**	766 (1)	
**22F (1)**	6403 (1)	
**23B (1)**	8886 (1)	
**17F (1)**	739 (1)	
**28A (1)**	494 (1)	
**13 (1)**	71 (1)	
**7C (1)**	737 (1)	

**Table 4:** New STs and their relationship with serotypes and STs in the database at MLST website (MLST database – accessed 19^th^ October, 2013).

Period*	ST	Serotype	SLV ST**	Serotype	Country
pre	8800	19A	43	14, 19F	Many
			424	19F	Many
			773	14	Colombia
			1113	7F	Uruguay
			4860	14	Brazil
	8879	6A	5576	6A	Brazil
	8883	9N	66	9N and others	Many, including Brazil
	8886	23B	387	23F	Brazil
			2777	6C	Brazil
			5962	23B	Brazil
			4431	23B	Brazil
	8888	6A	759	6A	Brazil
			757	6A	Brazil
	8890	19F	763	19F	Brazil, South Africa
	8894	14	156	14 and others	Many including Brazil
			162	14 and others	Many including Brazil
	8897#	6B			
					
pre/post	8889	20	235	20 and 7F	Spain, Austria, Poland
			8816	N/A	UK
			1345	20	UK
post	8881##	14			
					
	8882	11A	3184		
	8884	19A	320	19A and others	Many including Latin America
	8885	8	404	8	Europe and Brazil
			1629	8	UK
			7847	8	Scotland
	8891	18A	5063	18C	Brazil
	8893	4	5670	4	Brazil
	8895	23F	242	23F	Many including Brazil
	8896	6B	4977	6B	Brazil

*Pre: pre-vaccination; Post: post-vaccination;

**More frequent ST and/or the ones isolated in Brazil;

# 8897 is DLV of STs 2781 (serotype 6B) and 8248 (serotype 6A/B), isolated previously in Brazil;

## 8881 is DLV of STs 9 (serotype 14) and 15 (serotype 14). The first ST was previously isolated in many countries, as the second one, which was also related to Brazilian isolates.

Among the 70 STs identified, 17 (24.3%) were new in the MLST website database, 10 of which were presented in our population as singletons and 7 grouped in CCs (Figure 2 and Table 4). Eight of the new STs were found prior to vaccination; 8 were isolated after PCV10 use and 1 was associated with both periods (ST8889). New alleles were found in 4 of the 17 new STs. Comparing our population to the entire MLST database, we observed 7 previously unreported combinations of STs/serotypes, suggesting possible capsular switching; 5 (83.3%) were associated with non-PCV10 serotypes (Table 5).

**Table 5:** Isolates representing possible capsular switching associated with pre-existing STs, considering MLST database (accessed 19^th^ October, 2013).

Our Study						Previous studies		
ST	No. isolates	Period	Age (yrs)	Serotype	Site	Country	Serotype	Site
4967	1	Pre	37	19A	Blood	Brazil	23F	ND*
5406	1	Pre	<1	23F	Blood	Brazil	29	blood
	1	Post	80	9A	Blood			
166	1	Pre	<1	14	Blood	Many**	6B, 9, 9A, 9V, 11, 11A, 19A	Invasive and non- invasive
2104	2	Pre	49	35A	pleural	Spain	19F	blood
71	1	Pre	66	13	Blood	United Kingdom	22	CSF

*ND: Not defined;

**Taiwan, Korea, South Korea, Malaysia, France, Germany.

Three hundred and twenty two *S. pneumoniae* were viable for susceptibility testing. Overall, 42.8% (n = 138) had MICs greater than 0.06 µg/mL for penicillin (meningitis breakpoints) with a great ST variability (42 STs). Isolates with ceftriaxone MICs greater than 0.5 µg/mL (18.6%; n = 60) had a similar variability, with 15 STs identified. Considering non-meningitis breakpoints, 2 and 15 pneumococci were non-susceptible to penicillin (MIC = 4 µg/mL) and ceftriaxone (MIC = 2 µg/mL), respectively. Only one isolate from an adult (34 years old) was resistant to levofloxacin (MIC = 16 µ/mL), which was serotyped as 23F and sequence typed as ST4967. Non-susceptibility to erythromycin was observed in 7.9% (18/228) (among these, 11.1% (2/18) were intermediate and 88.9% (16/18) presented full resistance). The characteristics of these isolates, including STs and serotypes, are presented in Table 6. Despite the association of various distinct STs with the resistant isolates, three major CCs were present among these pneumococci (CC320, CC156 and CC338), grouping 74.3% of them. Serotypes 14 (n = 14) and 19A (n = 7) represented 58.3% of this specific population. Despite its association with resistant pneumococci, serotype 19A (n = 11) was also associated with susceptible isolates and represented a considerable genetic diversity (including two new STs): ST 2878 (n = 4), ST 320 (n = 2), ST 4967 (n = 1), ST 199 (n = 1), ST 733 (n = 1), ST 8800 (n = 1; new) and ST 8884 (n = 1; new).

**Table 6:** Characteristics of pneumococci recovered from IPD showing non-susceptibility to erythromycin, levofloxacin, ceftriaxone and penicillin.

Serotype (n)	ST (n)	CC	Susceptibility profile (µg/mL) (n/genotype)*
14 (11)	156 (5)	156	CRO = 2 (4)
			CRO = 2; ERY = 8 (1/*ermB*−; *mef*(A)+)
	13 (5)	singleton	ERY = 8 (5/*ermB*−; *mef*(A)+)
	370 (1)	156	CRO = 2 (1)
	8881 (1)	singleton	ERY = 8 (1/*ermB*−; *mef*(A)+)
19F (5)	271 (4)	320	ERY>32 (2/*ermB*+; *mef*(A)−)
			ERY>32; CRO = 2 (2/*ermB*+; *mef*(A)+)
	8890 (1)	320	ERY>32 (1/*ermB*+; *mef*(A)+)
19A (3)	320 (2)	320	ERY>32; CRO = 2 (1)
			ERY>32; PEN = 4; CRO = 2 (1)
	8884 (1)	320	ERY>32; PEN = 4; CRO = 2 (1/*ermB*+; *mef*(A)+)
23F (2)	4967 (2)	338	ERY = 0.5 (1/*ermB*−; *mefE*+)
			LEV = 16 (1/Ser^81^-Phe in *gyrA*; Ser^79^-Phe and Lys^137^-Asn in *parC*.)
3 (1)	180 (1)	singleton	ERY = 0.5 (1/*ermB*+; *mef*(A)−)
4 (1)	770 (1)	singleton	ERY = 0.5 (1/*ermB*−; *mef*(A)+)
6C (1)	172 (1)	338	CRO = 2 (1)
9A(1)	156 (1)	156	CRO = 2 (1)

*Genotype for erythromycin and levofloxacin resistance only; Penicillin (PEN), ceftriaxone (CRO), erythromycin (ERY) and levofloxacin (LEV) MICs.

One isolate was resistant to levofloxacin (1/322, 0.3%), and sequencing of *gyrA* and *parC* identified point mutations, Ser^81^-Phe in *gyrA* and Ser^79^-Phe and Lys^137^-Asn in *parC*. Resistance to macrolides was determined for 311 isolates and 8.0% (25/311) were non-susceptible (2 intermediate and 23 fully resistant), which was associated with *mef*(A) (11/25; 44.0%), *ermB* (9/25; 36.0%) and both *ermB* and *mefA* (5/25; 20.0%). All *ermB* positive isolates were also resistant to clindamycin (MIC≥1 µg/mL), regardless of *mefA* occurrence.

## Discussion

The present study generates important data about the molecular epidemiology of circulating pneumococci in Brazil. Most studies previously published from Brazil have not focused on evaluating the molecular epidemiology of the pneumococcal population or, if they have, it has related only to pneumococci from carriage [23]. To our knowledge, only two studies present characteristics of isolates recovered from patients with IPD in Brazil. Zemlicková and co-workers (2005) [24] evaluated invasive pneumococci from five different Latin American countries, including only 41 isolates from Brazil. The second study characterized only meningitis isolates and molecular analysis was focused on a specific population of 107 *S. pneumoniae* resistant to penicillin [25]. In our study, we included pneumococcal isolates from all invasive specimens over a six-year period regardless of age or antibiotic susceptibility to enable us to generate a more comprehensive scenario of molecular characteristics of pneumococci associated with IPD in our region and country.

This study was not designed to evaluate the efficacy of PCV10. However, we understand our data are relevant to provide an initial assessment of the impact of PCV10 use in Brazil. After the introduction of PCV10, a significant decline in hospitalizations for pneumonia was noted in many Brazilian cities, but not in Porto Alegre. This difference may in part be explained by the lower vaccination uptake (∼80%) compared to the other cities (>90%), as well as the delay in start date for vaccine use in this city [14]. Two years after the introduction of PCV10 our data suggest that this region seems to still have a transitional pneumococcal population and accumulating more recent data post 2012 would permit a better understanding on vaccine impact. The absence of PCV10 influence on the serotype distribution during the study period may be justified by the characteristics of our population, which was mainly composed of adults. Indeed, as we only evaluated two years post-vaccination (and considering the lower vaccine uptake observed in Porto Alegre), there may not have been sufficient time to observe the herd protection effect in this study population. Also, the very low number of isolates from children ≤5 years of age (38 patients) and the generally small number of isolates per year may have compromised statistical analysis. A well-designed case-control study [26], performed only with children and focusing in evaluating effectiveness of vaccine in Brazil recently demonstrated that PCV10 prevents invasive disease caused by vaccine serotypes. However, evaluation of the serotype distribution showed that serotypes 14, 6B, 23F, 18C and 19F remain among the most frequent serotypes causing invasive disease two years after vaccine introduction.

Although a few STs (ST53, ST156, ST180, ST191, ST218, ST770, ST4967 and ST8889) represented almost half of the isolates, our population presented a high degree of genetic diversity in both pre- and post-vaccination periods (Simpson’s index around 95%), which is similar to data from some reports [27], [28] but higher than others [29]. Several serotypes were especially clonal and primarily associated with one ST, such as 7F (ST191) and the non-PCV10 serotypes: 3 (ST180), 12F (ST218), 8 (ST53) and 20 (ST8889).

Serotype 3 has been associated with increased mortality in different regions [30], [31], [32]. Inverarity et al. (2011b) [33] demonstrated that mortality was strongly associated with ST180. The high frequency of isolation of this serotype (and ST) and its relation with mortality necessitates continued surveillance to monitor for increases in this serotype post-PCV10 as protection against serotype 3 may be an important reason to consider the use of PCV13 in our region.

Serotype 12F has been shown to cause outbreaks in human populations with identifiable risk factors [34], [35], [36], [37]. This serotype has a high case:carriage ratio (CCR), i.e., it is a hyper invasive serotype, and is rarely found in the nasopharynx [23], [35], [36], [37]. In our population, this serotype was among the most frequent serotypes, presented a highly clonal occurrence and was exclusively distributed among adults.

All but two pneumococci from serotype 8, which also presents high CCR, were characterized as ST53 (PMEN clone Netherlands^8^-33), a well-recognized virulent clone [38], [39], [40] The remaining isolates were identified as ST404 and as a new one, ST8885, a SLV of ST404, which has already been isolated in different European countries and previously in Brazil [41] (Table 4).

Although somewhat controversial, serotype 7F also appears to be associated with high case-fatality [7], [9]. Some authors have observed serotype 7F as one of the main serotypes associated with replacement following PCV7 introduction, through clonal expansion [28], [29]. Pichon and co-workers (2013) [28] demonstrated ST191 (serotype 7F) as the most prevalent clone causing meningitis 3 years after the introduction of PCV7 in England and Wales. From reported studies, serotype 7F seems to be very rare in the nasopharynx of Brazilian children [23], [42], [43]. All but 2 (ST218) of our serotype 7F invasive pneumococci belonged to ST191, and the ST218 isolates are possibly capsular switch variants of serotype 12F.

ST8889, a new ST reported in this study, was exclusively related to serotype 20, and appears to be well established in Brazil, being detected over the 6 year period of this study. Seotype 20 is not part of any conjugated vaccine, although it is among the 23 selected serotypes of the polysaccharide vaccine. and little is known about its virulence and invasive characteristics. In the present study, this serotype was exclusively associated with IPD in adults, both before and after vaccination. ST8889 is a single locus variant (SLV) of three other STs recovered in Europe (Table 4): ST235, ST1345 and ST8816 and is a double locus variant (DLV) of an ST described in Brazil in 2001, from a meningitis case (ST762). Of note, serotype 20 seems to rarely colonize Brazilian children [23], [43], [44].

ST770 has already been described in Brazil as serotypes 4 and 23F. Interestingly, ST770 is a DLV of ST758, observed in two Brazilian meningitis cases in 2001, both described as serotype 20. Similarly, ST770 is a SLV of other STs from meningitis described in Brazil: ST8169 (serotype 4) in 2001 and ST772 (non-typable) in 2006, as demonstrated by data recovered from the MLST database.

CC156 has frequent occurrence around the world [45], including Latin America and Brazil [23], [24], [25]. ST156 (PMEN clone Spain^9V^-3), a member of this CC and the most frequent ST among our population with both serotypes 9V and 14, is globally associated with important resistance profiles, including in Brazil [25]. We observed one isolate (recovered after vaccine introduction) with ST156 classified as serotype 9A, which has been previously described in the MLST database (Qatar and Lebanon, in 2006). We did not observe any PCV10 effect on this CC, as the occurrence before and after vaccination was quite similar in our population.

Our study shows a high genetic diversity including a considerable number of STs not previously reported. Moreover, the results show new capsular and clone combinations (suggesting capsular switching). Pneumococci with new combinations were mostly recovered during the pre-vaccine period, highlighting the natural diversity in the pneumococcal population and that the occurrence of factors other than selective pressure from vaccination encourages diversity.

The increase of serotype 19A in both carriage and invasive disease after PCV7 introduction is well documented [46]. However, this serotype also increased in regions without vaccine selective pressure, suggesting the participation of other factors, such as dissemination of some specific clones and antimicrobial pressure [47] in this process. Serotype 19A is frequently associated with ST199 and ST320, a DLV of ST236 (PMEN clone Taiwan^19F^-14) [46], [48]. ST320 is globally disseminated and strongly associated with penicillin resistance [47], [49], [50], [51]. Recently, it was observed that the genetic background of ST320 provides advantages associated with improved colonization in the nasopharynx when compared to ST199 [48]. This advantage may be responsible in part for the rapid shift of ST199 to ST320 in USA soon after PCV7 [47]. However, in Germany, a country with low antibiotic pressure, the increase in 19A was attributed mainly to CC199 which increased and not CC320, suggesting multiple factors in play [52].

Both, ST320 and ST199 were recognized in the present work, although in low numbers. Despite the genetic diversity observed among serotype 19A in our population, ST2878 was the most frequent (36.4%). This ST had been documented in Brazil several other times and in different regions, with the first isolate recovered in 1998 (MLST database). Our study demonstrates the presence of ST2878 up to 2012, suggesting the widespread dissemination of this clone in Brazil.

CC320/271and CC156 were common among our penicillin non-susceptible pneumococci Although the proportion of isolates that were CC320 was considerably lower than for CC156, MIC values for penicillin among the CC320 isolates were higher (range 1 to 4 µg/mL). ST320 (serotype 19A) and ST271 (serotype 19F) are two well-recognized clones in CC320/271, while ST8884 (serotype 19A), a SLV of ST320, is newly described in this study. Our isolates belonging to ST8884 were multidrug-resistant, with resistance to tetracycline, erythromycin (with *ermB* and *mefA* genes) and non-susceptibility to β-lactams. Systematic molecular surveillance may be important to generate more data about this ST and its dissemination profile.

The prevalence of penicillin resistance seems to be variable, with reported rates as low as 0% [53], [54], [55] and as high as 27.9% [25], [54] in Brazil. It is important to point out that previously published rates used the 2008 CLSI breakpoints to determine β-lactams susceptibility, and in this study, penicillin MICs ≥4 µg/mL were not observed. Non-susceptibility is mostly associated with serotypes 14, 19F, 23F, 6B and 19A [24], [25], [54] in Brazil. Although Zemlicková et al. (2005) [24] demonstrated a heterogeneous pre-vaccination population of penicillin-resistant pneumococci, Barroso et al. (2012) [25] suggested a major role of clone Spain^9V^-3 among pneumococci with this resistance. Even though this clone was commonly observed in our population, we did not observe an association with non-susceptible (MIC = 4 µg/mL) isolates, which were exclusively characterized as serotype 19A.

This study described pneumococcal population dynamics of isolates causing IPD in South Brazil, with a particular focus on the period after PCV10 implementation. Limitations of the study included few patients under five years of age (the target group for PCV10). In addition, given the relatively recent introduction of PCV10, our data reflect early effects of the immunization program. Continued surveillance and further studies need to be conducted to determine the full impact of vaccine in all age groups and to follow changes in pneumococcal population biology post-PCV10 introduction in Brazil.

## Supporting Information

Table S1 **Serotype distribution among invasive pneumococcal from 2007–2012.**
(DOC)
