# Supplementary material for: Serotypes and Genotypes of Invasive Streptococcus pneumoniae Before and After PCV10 Implementation in Southern Brazil
Source: PLoS One. 2014 Oct 30;9(10):e111129. doi: 10.1371/journal.pone.0111129 (PMC4214725; doi:10.1371/journal.pone.0111129)
Supplement: Table S1 — Serotype distribution among invasive pneumococcal from 2007–2012. (DOC) [file pone.0111129.s001.doc]

Table S1: serotype distribution among invasive pneumococcal from 2007-2012.

| **Serotype** | **N (%)** | **Pre*** | **Post**** | **OR^#^** | **95% CI** | **P** |
| --- | --- | --- | --- | --- | --- | --- |
| 14 | 37 (11.4) | 21 | 16 | 0.7992 | 0.3998-1.5976 | 0.5260 |
| 3 | 27 (8.3) | 17 | 10 | 1.0683 | 0.4728-2.4139 | 0.8738 |
| 4 | 23 (7.1) | 10 | 13 | 0.4534 | 0.1925-1.0682 | 0.0704 |
| 23F | 20 (6.2) | 12 | 8 | 0.9335 | 0.3706-2.3516 | 0.8840 |
| 7F | 19 (5.8) | 12 | 7 | 1.0760 | 0.4119-2.8106 | 0.8812 |
| 12F | 16 (4.9) | 8 | 8 | 0.6094 | 0.2227-1.6671 | 0.3348 |
| 9V | 16 (4.9) | 10 | 6 | 1.0439 | 0.3698-2.9467 | 0.9354 |
| 20 | 16 (4.9) | 11 | 5 | 1.3968 | 0.4736-4.1199 | 0.5448 |
| 19F | 13 (4.0) | 10 | 3 | 2.1404 | 0.5774-7.9334 | 0.2549 |
| 8 | 13 (4.0) | 5 | 8 | 0.3750 | 0.1199-1.1733 | 0.0919 |
| 19A | 11 (3.4) | 5 | 6 | 0.5085 | 0.1519-1.7029 | 0.2729 |
| 5 | 11 (3.4) | 8 | 3 | 1.6944 | 0.4409-6.5113 | 0.4426 |
| 6A | 10 (3.1) | 9 | 1 | 5.8429 | 0.7312-46.6925 | 0.0960 |
| 6B | 10 (3.1) | 5 | 5 | 0.6154 | 0.1745-2.1701 | 0.4502 |
| 11A | 8 (2.5) | 2 | 6 | 0.2003 | 0.0398-1.0087 | 0.0512 |
| 16F | 8 (2.5) | 6 | 2 | 1.8866 | 0.3747-9.4977 | 0.4414 |
| 18C | 7 (2.0) | 6 | 1 | 3.8351 | 0.4562-32.2393 | 0.2159 |
| 1 | 7 (2.0) | 6 | 1 | 3.8351 | 0.2265-6.9555 | 0.2159 |
| 9N | 6 (1.8) | 4 | 2 | 1.2551 | 0.4684-150.2134 | 0.7948 |
| 10A | 6 (1.8) | 6 | 0 | 8.3882 | 0.1942-18.3573 | 0.1485 |
| 6C | 4 (1.2) | 3 | 1 | 1.8883 | 0.0864-4.4673 | 0.5838 |
| 24F | 4 (1.2) | 2 | 2 | 0.6212 | 0.2278-86.8475 | 0.6362 |
| 15A | 3 (0.9) | 3 | 0 | 4.4481 | 0.0277-3.4444 | 0.3249 |
| 18A | 3 (0.9) | 1 | 2 | 0.3900 | 0.3278-86.8475 | 0.3398 |
| 23B | 3 (0.9) | 3 | 0 | 4.4481 | 0.0386-10.0532 | 0.3249 |
| 35F | 2 (0.6) | 1 | 1 | 0.6231 | 01505-66.3929 | 0.7388 |
| 35A | 2 (0.6) | 2 | 0 | 3.1612 | 0.0059-2.5875 | 0.4587 |
| 9A | 2 (0.6) | 0 | 2 | 0.1232 | 0.0386-10.0632 | 0.1777 |
| 22F | 2 (0.6) | 1 | 1 | 0.6231 | 01505-66.3929 | 0.7388 |
| 17F | 2 (0.6) | 2 | 0 | 3.1612 | 0.0059-2.5875 | 0.4587 |
| 38 | 2 (0.6) | 0 | 2 | 0.1232 | 0.0386-10.0632 | 0.1777 |
| 29 | 2 (0.6) | 2 | 0 | 3.1612 | 0.0059-2.5875 | 0.4587 |
| 13 | 1 (0.3) | 1 | 0 | 1.8872 | 0.0763-46.6910 | 0.6980 |
| 15B | 1 (0.3) | 1 | 0 | 1.8872 | 0.0763-46.6910 | 0.6980 |
| 34 | 1 (0.3) | 0 | 1 | 0.2070 | 0.0084-5.1210 | 0.3359 |
| 15C | 1 (0.3) | 1 | 0 | 1.8872 | 0.0763-46.6910 | 0.6980 |
| 28A | 1 (0.3) | 0 | 1 | 0.2070 | 0.0084-5.1210 | 0.3359 |
| 33F | 1 (0.3) | 1 | 0 | 1.8872 | 0.0763-46.6910 | 0.6980 |
| 7C | 1 (0.3) | 0 | 1 | 0.2070 | 0.0084-5.1210 | 0.3359 |
| NT | 3 (0.9) | 3 | 0 | 4.4481 | 0.2278-86.8475 | 0.3249 |
| **Total** | **325** | **200** | **125** |  |  |  |

*Pre-vaccination period; **post-vaccination period; ^#^OR: odds ration
